# Nectar in Plant Species *Fragaria vesca* L.

**DOI:** 10.3390/plants14182938

**Published:** 2025-09-22

**Authors:** Katja Malovrh, Jože Bavcon, Mitja Križman, Blanka Ravnjak

**Affiliations:** 1 Botanical Gardens of Ljubljana, Biotehnical Faculty, Universtiy of Ljubljana, 1000 Ljubljana, Slovenia; katja.malovrh@bf.uni-lj.si (K.M.); blanka.ravnjak@bf.uni-lj.si (B.R.); 2 Department of Analytical Chemistry, National Institute of Chemistry, Hajdrihova 19, P.O. Box 660, 1001 Ljubljana, Slovenia

**Keywords:** amino acids, nectar production, phenolic compounds, strawberry, sugars

## Abstract

*Fragaria vesca* L. is a common plant species in Slovenia. It flowers from May to July. Our study was conducted throughout the 2024 season in two locations at which we sampled nectar in *F. vesca* flowers. To take the nectar samples, we used microcapillaries. We studied *Fragaria vesca* nectar production and its composition (sugars, amino acids, and phenolic compounds) throughout the day. We had some problems with sampling nectar in the afternoon, which affected our research, since there were times during which we could not obtain any samples. *F. vesca* on average secreted 0.02 μL nectar per one flower sample. Our data show that nectar production is highest in the morning, nectar is hexose-dominant, and the time of day affects the sugar concentration, which reaches a maximum at noon. The most common amino acid in *F. vesca* nectar is proline, and the amino acid concentration is highest in the morning. Quercetin and rutin are common phenolic compounds in the nectar of *F. vesca,* and the concentration of phenolic acids is highest at noon, as bees are the most active in the spring when mornings are colder.

## 1. Introduction

Plants provide pollinators with food mainly in the form of nectar, as well as pollen and honeydew [1]. Nectar primarily functions to attract pollinators and serves as a nutritional resource for them [2,3]. Nectar is secreted by nectaries, which are specialized glands that possess specialized cells and tissues dedicated to the regular production and secretion of nectar [3,4,5]. A nectary consists of three main components: the epidermis, specialized parenchyma, and a vascular system. Nectar is secreted through the epidermis, while the parenchyma either produces or stores metabolites; water and nutrients are transported to the parenchyma via the vascular system [4,5,6]. In the Rosaceae family, nectar is secreted through modified stomata—pores in the epidermal cells—whose number and location may vary among species within the family [7,8].

Nectar can contain up to 90% water, as much as 70% sugars, nitrogen compounds, organic acids, pigments, essential oils, vitamins, and mineral substances. The most common sugars found in nectar are sucrose, fructose, and glucose. The ratio of sucrose to monosaccharides depends on the presence and activity of various enzymes, primarily invertase, an enzyme responsible for the catalytic hydrolysis of sucrose into fructose and glucose. This hydrolysis increases the osmotic potential of the nectar, thereby enabling it to absorb more water. This means that sucrose no longer predominates, while the nectar is also more diluted. As a result, studies yield different composition outcomes. Depending on the ratio of glucose, fructose, and sucrose, nectar can be classified into basic types: sucrose-dominant, sucrose-rich, hexose-rich, and hexose-dominant [1,9]. Nectar may also contain lower concentrations of monosaccharides such as mannose, arabinose, xylose, and disaccharides such as maltose and melibiose [1,10]. Sugar, and consequently nectar, is also an excellent medium for microbial growth. With the visits of pollinators collecting nectar, these microbes can be transmitted from flower to flower and influence the nectar by increasing the proportion of certain sugars. Yeasts, for instance, increase the total sugar content and the percentage of fructose, while the percentage of sucrose decreases [11]. Percival [12] studied the sugars in the nectar of a large number of angiosperms. She found that the composition may also be influenced by the shape of the flower. Plants with deep and tubular flowers produce nectar in which sucrose predominates, whereas those with shallower flowers produce nectar dominated by glucose or fructose.

The next most abundant compounds in nectar are amino acids. Although they make up less than 5%, amino acids are believed to be present in the nectar of all plant species, but due to their low concentration, they are difficult to detect [6,13,14,15]. Nectar contains both essential and non-essential amino acids [16]. Amino acids, especially proline—which insects require in the early stages of development—play an important role in maintaining good muscle condition, which is necessary for movement [17]. Proline is the dominant amino acid in the hemolymph of insects, including bees.

About ten different phenolic compounds can be found in nectar, depending on the plant species. Rutin is believed to be the most common phenolic compound in nectar [18]. It is very important for bees, as it has a protective effect against certain insecticides [19]. The role of phenolic compounds is either to attract or repel pollinators and herbivores [20].

The literature presents conflicting views on whether the genus *Fragaria* produces nectar. Some sources state that *Fragaria* species do not have nectar [21], while others report that they do [22]. Given that this is a common genus in Slovenian flora, we were interested in whether it truly offers only pollen to pollinators or also nectar. In the nectar, we analyzed the content of certain sugars, amino acids, and phenolic compounds, and examined which part of the day has the highest nectar secretion. 

## 2. Results

### 2.1. Nectar in Fragaria vesca Flowers

We sampled nectar from the species 96 times; however, the sampling process was not always successful, as a detectable amount of nectar was not always obtained. In the morning, 97% of samples were successfully collected, at noon, 31% were successfully collected, and in the afternoon, only 12.5% were successfully collected.

On average, the species secretes 0.02 μL of nectar. Table 1 shows the average mass of individual compounds in the nectar of this species. Sugars dominate the nectar composition, while phenolic compounds and amino acids are present in considerably lower amounts.

### 2.2. Nectar Secretion in Fragaria vesca

**Figure 1:**
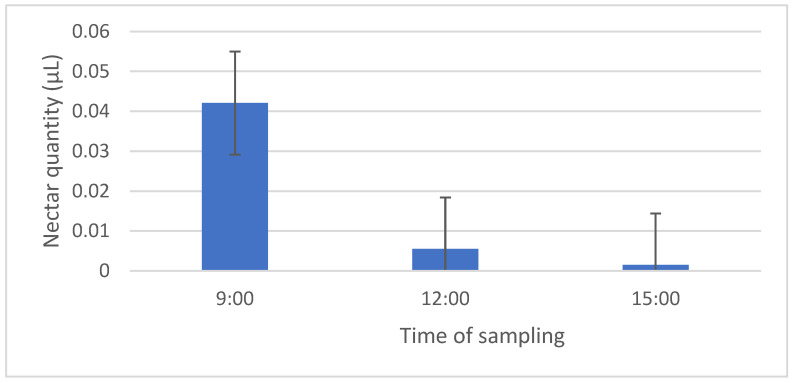
Average amount of nectar secreted per flower of *Fragaria vesca*. (*N* = 32 per hour).

The highest amount of nectar was secreted in the morning hours (0.04 μL), while significantly less was secreted at noon and in the afternoon (Figure 1). At noon, the average secretion was 0.0055 μL, and in the afternoon, it was only 0.0015 μL of nectar. The lack of samples at noon and in the afternoon could affect the lower average amount of nectar.

### 2.3. Three Main Sugars in Fragaria vesca Nectar

We found that in the nectar of *F. vesca*, regardless of the time of day, sucrose was consistently the least abundant sugar. 

**Figure 2:**
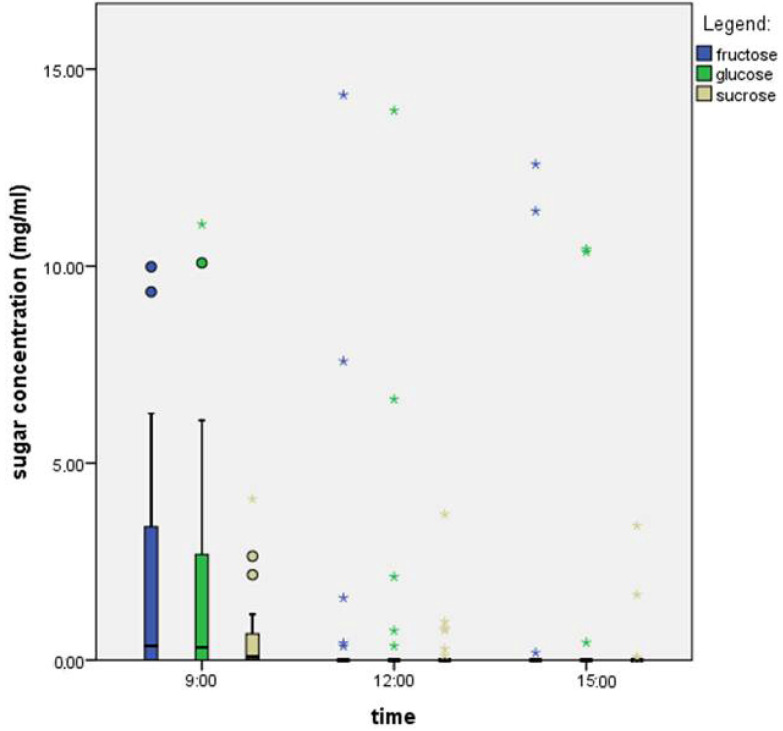
Sugar concentrations throughout the day in the nectar of a single flower of *F. vesca*. The boxplot shows the median (line within each box), quartiles, and standard deviation (vertical lines). *N* = 32 nectar samples per hour. Circles present mild outliers and stars present extreme outliers.

Glucose was the most abundant in the morning and at noon, while fructose predominated in the afternoon (Figure 2). Using the Kruskal–Wallis test, we identified statistically significant differences between the time points (SAH: 0.00, GLU: 0.000, and FRU: 0.000). The Mann–Whitney U test confirmed statistically significant differences in the concentrations of all three sugars between 9:00 and 12:00, as well as between 12:00 and 15:00, which means that the time of day affects the sugar concentration.

We found that the total sugar concentration was highest at noon—8.70 mg/mL (Table 2)—despite a considerable number of samples lacking nectar. The lowest sugar concentration was recorded in the afternoon—3.22 mg/mL. The glucose and fructose concentrations were similar, while sucrose was present in significantly lower amounts.

Nectar is dominated by three sugars: sucrose, glucose, and fructose. Based on the ratio of sucrose concentration to the combined concentration of fructose and glucose, nectar can be classified into four groups: hexose-dominant (S/(G + F) < 0.1), hexose-rich (S/(G + F) = 0.1–0.49), sucrose-rich (S/(G + F) = 0.5–0.99), and sucrose-dominant (S/(G + F) ≥ 1) [23]. Based on our calculation (0.1/(0.5 + 0.5) = 0.1), the nectar of *F. vesca* is classified as hexose-dominant. Although the time of sampling influences the total sugar concentration—with the highest values recorded at noon—it does not affect which sugar is dominant.

### 2.4. Selected Amino Acids in Fragaria vesca Nectar

**Figure 3:**
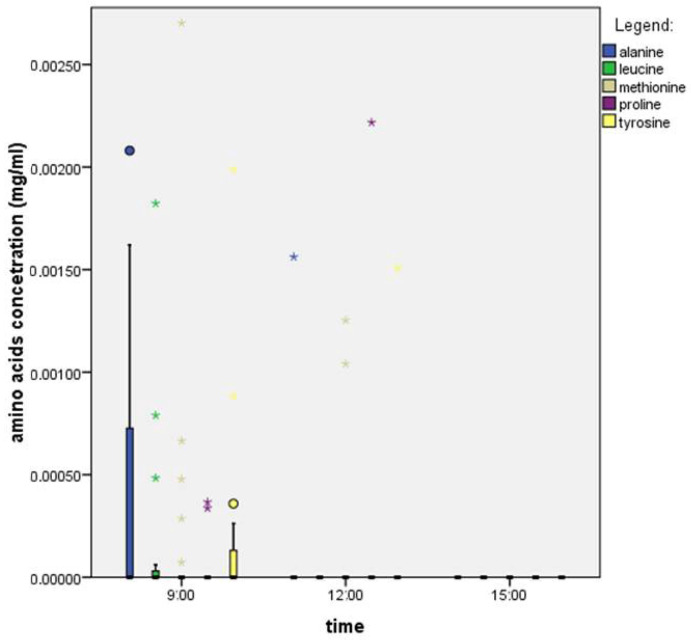
Amino acid concentrations throughout the day in the nectar of a single flower of *F. vesca*. The boxplot shows the median (line within each box), quartiles, and standard deviation (vertical lines). *N* = 32 nectar samples per hour. Circles present mild outliers and stars present extreme outliers.

We found that in the nectar of *F. vesca*, the most abundant amino acid throughout the day was proline, while methionine was the least abundant among those analyzed. As with sugars, the concentration of amino acids in this species also declined significantly over the course of the day (Figure 3). We used the Kruskal–Wallis test, which showed that there were statistically significant differences between time points in amino acid concentrations (PRO: 0.153, LEV: 0.081, MET: 0.091, ALA: 0.036, and TIR: 0.090). This indicates that the time of sampling influenced the concentration of alanine. The Mann–Whitney U test showed that there were statistically significant differences in alanine concentration between 9:00 and 15:00.

In calculating the average amino acid concentrations (Table 3), we found that the total concentration of amino acids was highest in the morning (0.057 mg/mL) and decreased over the course of the day—at noon, it was 0.015 mg/mL, and it was 0.010 mg/mL in the afternoon. The time of sampling influenced the concentration of amino acids, but it did not affect the dominant amino acid throughout the day, which was proline.

### 2.5. Selected Phenolic Compounds Fragaria vesca Nectar

**Figure 4:**
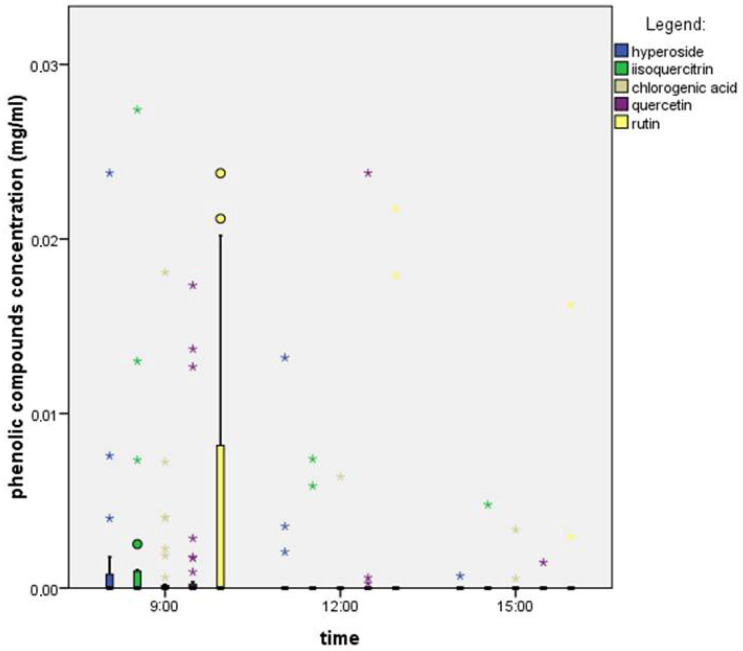
Phenolic compounds’ concentrations throughout the day in the nectar of a single flower of *F. vesca*. The boxplot shows the median (line within each box), quartiles, and standard deviation (vertical lines). *N* = 32 nectar samples per hour. Circles present mild outliers and stars present extreme outliers.

We found that in the nectar of *F*. *vesca*, rutin was the most abundant in the morning and at noon, while quercetin dominated in the afternoon (Figure 4). We used the Kruskal–Wallis test, which showed that there were statistically significant differences between time points of amino acid concentrations (KLO: 0.032, RUT: 0.021, IZO: 0.027, HIP: 0.022, and KVE: 0.029). The Mann–Whitney U test showed that there were statistically significant differences in the concentration of phenolic compounds between 9:00 and 15:00.

In calculating the average concentrations of phenolic compounds (Table 4), we found that the total concentration was highest at noon (0.054 mg/mL) and in the morning (0.043 mg/mL), and it decreased over the afternoon to 0.0067 mg/mL. The time of sampling influenced both the concentration of phenolic compounds and the dominant phenolic compound. Rutin was predominant in the morning and at noon, while quercetin became dominant in the afternoon.

## 3. Discussion

*Fragaria vesca* is a common plant species along forest edges and blooms from May to June [24,25]. There has been some debate about whether nectar is present in *F. vesca*, as some sources claim the species does not produce it [21]. We found that the species does secrete nectar, but the quantities are small.

Nectar is secreted on the gynoecium [26], which is more exposed to external factors and therefore more susceptible to evaporation [22]. As a result, we experienced difficulties in sampling at noon and in the afternoon, as nectar was often not retrievable. Despite this, the average sugar concentration was highest at noon, which likely indicates that evaporation occurred, resulting in more concentrated nectar. In some *Fragaria* species, nectar secretion is said to occur only during the first five days, which is related to the sequential development of floral organs, in which the female part develops first, followed later by the male part [22]. The reduced nectar secretion at noon and in the afternoon could also be due to flower damage, as the use of capillaries can be challenging for smaller flowers. Although the flower of *F. vesca* is open, rough handling or excessive pressure from the capillary on the gynoecium can sometimes cause damage to the flower. Some researchers [27] recommend the use of microcapillaries primarily for deep flowers; however, in our study, we found this method to be suitable even for shallow flowers such as those of *F. vesca*. From the perspective of subsequent analyses, it also proved to be more appropriate and accurate than other methods (e.g., refractometry, flower rinsing, or washing). In general, nectar secretion is reported to be highest between 10:00 and 12:00 [28,29,30,31,32,33,34]. Nectar production decreases from morning to afternoon [32,35], which was also evident in our study. A changing amount of nectar secretion is related to bee activity. Bees are more active around noon [36]. Also, other abiotic factors, such as temperature, can affect nectar secretion; for example, low humidity and high temperatures could lead to lower nectar secretion [6,29].

In general, nectar is dominated by three sugars: sucrose, glucose, and fructose. We determined that the nectar of *F. vesca* is hexose-dominant and contains very little sucrose. No studies on the sugar composition of nectar in *F. vesca* have been conducted so far; however, research has been carried out on cultivars of the genus *Rubus*, which, like our study species, also belongs to the Rosaceae family. It was found that members of the genus *Rubus* have hexose-dominant nectars with a very low proportion of sucrose. Of the six cultivars analyzed, only two contained sucrose [10]. Similar findings were reported for the genus *Prunus* [37]. Percival [12] linked a high proportion of hexoses in nectar to flower morphology, suggesting that more open flowers, such as those of *F. vesca*, tend to have hexose-rich nectar. Despite nectar secretion being highest in the morning, the sugar concentration was highest at noon. This is likely due to higher air humidity in the morning, which can result in a greater volume of nectar but a lower sugar content—making it more diluted [1,38]. Similar results were observed in studies of nectar from the genera *Crataegus* and *Prunus*, in which the highest sugar concentrations were also recorded at noon [38,39]. We collected a small quantity of nectar, which may be due to its viscosity, as viscous nectar is more difficult to extract with a capillary [40].

Phenolic compounds are considered one of the main groups of secondary metabolites in plants [36]. Plants produce phenolic compounds mainly for growth, development, and protection. They are particularly important when plants are under biotic or abiotic stress. At such times, their levels increase and they serve as protection against stress factors [23,41]. Some plant secondary metabolites are also important for insects, as they affect their nervous system by binding to neuronal receptor proteins, thereby influencing their behavior. They can enhance insect memory, assist in foraging, and offer protection against parasitic infections [42]. Knowledge of the phenolic compounds in nectar is also important from a human perspective, as nectar is the raw material for honey production. Phenolic compounds also have medicinal effects on human health [43]. In our study of the phenolic compounds in nectar, we focused on five compounds: rutin, hyperoside, chlorogenic acid, quercetin, and isoquercitrin/quercitrin. Rutin was the dominant phenolic compound. Other studies on various plant species within the Rosaceae family have also found rutin to be the most common [44,45]. It is the most important phenolic compound, as it can protect bees from insecticides [19]. Chlorogenic acid was detected in the smallest amounts. It can even be toxic to bees and acts as a repellent [46,47] although low levels of chlorogenic acid are not truly toxic to bees [48,49]. In the afternoon, however, quercetin began to predominate. Quercetin affects the bee nervous system and enhances their memory, which in turn helps them locate foraging areas [50]. Under normal conditions, queen pheromones suppress the reproductive potential of worker bees and prevent the development of new queens. However, feeding workers with nectar rich in quercetin stimulates ovary development and the formation of queen cells, thereby promoting queen rearing—an outcome that is undesirable for beekeepers, as it may lead to colony disruption [51]. The concentration of phenolic compounds was highest at noon—coinciding with the time during which the bees were most active [36,52].

Nectar contains both essential and non-essential amino acids [16]. In our study, we focused on five amino acids that are important for pollinators and are commonly found in nectar. We found that proline was the most abundant amino acid in the majority of the plant species analyzed. Bees are believed to actively seek out flowers with higher concentrations of proline [53]. Proline is important in the early developmental stages of insects, as it plays a key role in maintaining their physical condition and in the formation of muscle fibers essential for locomotion [17]. Other researchers have similarly found that proline is the most abundant amino acid, accounting for more than 50% of all amino acids present [10,13]. However, the high proportion of proline could also be partially due to the presence of pollen in the nectar, especially considering the flower morphology of our flowers [50]. We cannot confirm that the concentration of proline was not affected by pollen. Methionine was detected in the lowest concentrations. It actually serves as a defense compound for the plant and may even be toxic to certain insects [14]. Tyrosine was also present in our samples, though at lower concentrations, consistent with the findings of Bertazzini and Forlani [13]. The concentration of amino acids was highest in the morning, which coincided with peak bee activity [52].

For further analysis and more precise results, we recommend collecting flowers that are the same age to see if the age of the flowers also affects nectar secretion.

## 4. Materials and Methods

### 4.1. Genus Fragaria

The flowers of species in the Rosaceae family are often large, colorful, bisexual, and star-shaped. The stamens are usually numerous and arranged in whorls [24,54]. In Slovenia, three species grow naturally (*F. vesca* L., *F. moschata* Duchesne, and *Fragaria viridis* Duchesne), along with one hybrid, *F. × ananassa* Duchesne [24]. The first two species are widespread throughout Slovenia [55].

**Figure 5:**
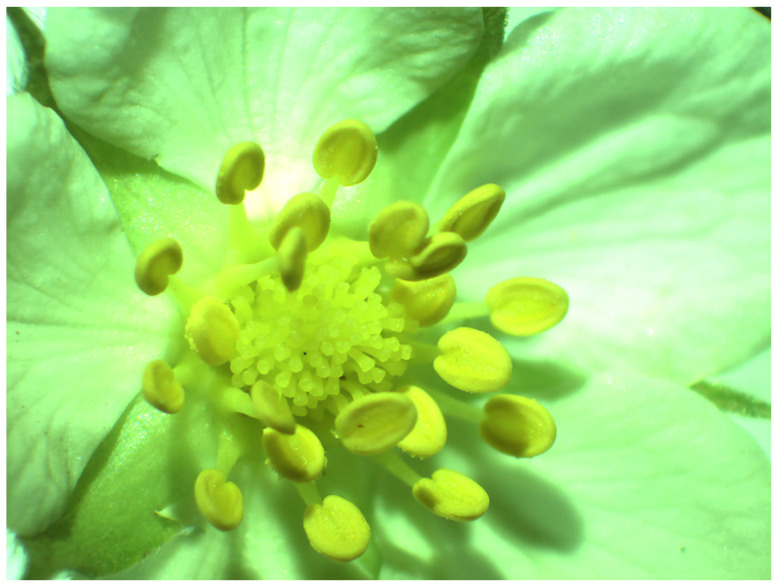
*F. vesca* flower (10× magnification).

We studied the nectar of the species *Fragaria vesca*. It is the most widespread species of its genus in Slovenia [25]. Its habitats include forest edges, clearings, and rocky grassy slopes, ranging from lowlands to the subalpine belt. It blooms between May and June. It has five white petals and numerous carpels (Figure 5). The plants are monoecious, and all flowers have normally developed stamens and pistils. Each individual plant has 4 to 8 flowers [24]. The flower stalks have mostly appressed hairs. To aid species identification, the bract of the inflorescence—similar to the true leaves and always serrated—is also helpful [25]. Nectar is secreted on the gynoecium (Figure 5), which, in some species of the genus *Fragaria,* is not always present [26].

### 4.2. Sampling Locations

**Figure 6:**
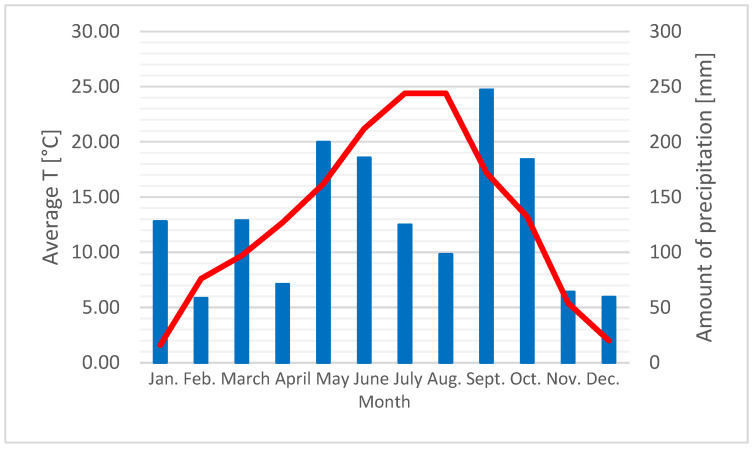
Average annual temperature and precipitation in 2024 [56]. Red line represents average temperature, and blue columns represent amount of precipitation.

Ljubljana is situated in the central part of Slovenia and has a moderately continental climate. Winters often bring temperatures below freezing, while summers are warm. Precipitation is more frequent in spring and autumn [56]. Slovenia lies at the intersection of four biogeographic regions: Alpine, Dinaric, Pannonian, and Sub-Mediterranean. It is located in a moderately warm climatic zone. Due to Slovenia’s transitional position, the influence of the following phytogeographic regions can be felt: Alpine, Dinaric, and Sub-Mediterranean. Average annual temperature and precipitation are shown in Figure 6.

Nectar sampling was always carried out using individuals growing at the forest edge. Sampling was conducted in April 2024. We sampled the species at two locations: one on the outskirts of Ljubljana (location 1) and the other within the city of Ljubljana (location 2).

**Figure 7:**
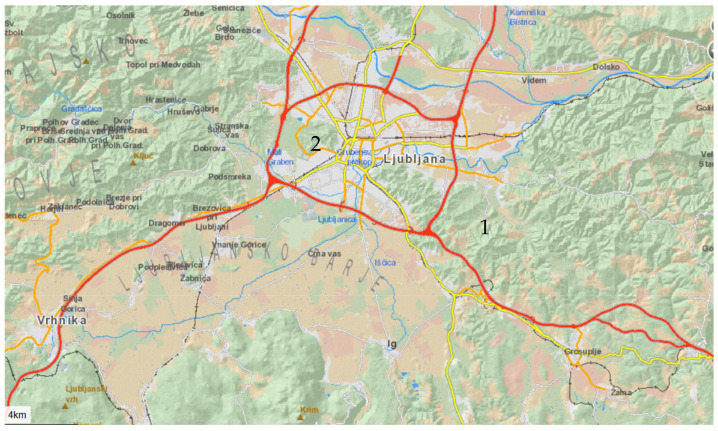
Map of sampling locations (1—Orle, 2—Večna pot Biological Center).

Location: The path to Orle (Figure 7)

The path to Orle is located at an elevation of 330 to 400 m above sea level. The site was quite diverse in terms of habitat types, with both marshy areas and even dry grasslands visible. From the bottom of the slope up toward Orle, the terrain was quite moist due to streams flowing down from the hill. The stream and meadows were surrounded by beech forest. On one side, the meadow was marshy and shaded; on the other, it was sun-exposed and dry. As one moved uphill toward Orle, the forested area gradually decreased, transitioning into dry grasslands.

Location 2: Večna pot Biological Centre (Figure 7)

At the location, situated at 295 m above sea level, a wet meadow dominated by marsh plant species prevailed. The forest edge was composed of various shrub species (*Crataegus monogyna* Jacq. and *Prunus spinosa* L.). The soil, even in the forested part, was frequently flooded, especially in spring.

### 4.3. Nectar Sampling

In our study, we pre-covered the plants with fleece the day before sampling. Since nectar production and persistence are also influenced by rain [27], we took samples only on dry days. Nectar was collected at three different times in one day: at 9:00, 12:00, and 15:00 over eight days. We sampled the same four individuals throughout the day. In total, we collected 96 nectar samples. We further analyzed 45 samples due lack of samples.

We used the microcapillary sampling method [57,58,59], in which nectar is drawn by capillary action [40]. For sampling with capillaries, we used 1 µL microcapillaries (Vitrex Medical, Herlev, Denmark). When handling them, we wore latex gloves. After drawing the nectar, we immediately measured its height in the microcapillary tube. Since the volume of the capillary tube was known, we were able to calculate the volume of nectar collected from a single flower directly in the field. The microcapillary tubes with the collected nectar were placed in centrifuge tubes and stored in a −20 °C freezer, where they remained until further analysis.

The samples stored in the freezer were thawed and transferred into vials for further analysis. The samples collected with microcapillaries were diluted in 150 µL of water per sample in 2 mL centrifuge tubes. The centrifuge tubes were then placed in a centrifuge for two rounds of 1 min each. After final centrifugation (3 min), the liquid was transferred to an HPLC vial.

### 4.4. Analysis of Main Sugars

Standards of the three main sugars—sucrose, glucose, and fructose (Merck Millipore, Darmstadt, Germany)—were prepared by dissolving 100 mg of each sugar in 10 mL of deionized water (MilliQ, Merck Millipore). The standards were then diluted to obtain a working standard solution with a concentration of 1 mg/mL for each sugar. The diluted nectar samples were analyzed using a UHPLC Vanquish system (Thermo Scientific, San Jose, CA, USA) equipped with a charged aerosol detector (CAD) and Chromeleon 7.2 SR4 data acquisition software (Thermo Scientific). The separation column used was a Nucleogel Sugar Ca with dimensions of 300 mm × 6.5 mm i.d. (Macherey-Nagel, Düren, Germany) at a temperature of 90 °C. The mobile phase was water under isocratic conditions (the composition of the mobile phase remained constant throughout the analysis), with a flow rate of 0.7 mL/min. The temperature of the CAD detector source was set to 90 °C. The analysis time was 13 min. Sample vials were thermostatted at 10 °C, and the sampler rinse solution was water. Injection volumes were 5 µL for standard solutions and 15 µL for samples.

### 4.5. Analysis of Selected Amino Acids

The standard amino acid solution was purchased from Sigma-Aldrich (St. Louis, MO, USA) and contained 10 µg/mL of each analyzed amino acid (proline, methionine, leucine, isoleucine, tyrosine, and alanine). The diluted nectar samples were analyzed using an HPLC Accela 600 system (Thermo Scientific, San Jose, CA, USA) coupled with a TSQ Quantum mass spectrometer equipped with an electrospray ionization (ESI) source (Thermo Scientific) and Xcalibur 2.1 data acquisition software (Thermo Scientific).

Detection was performed in selected ion monitoring (SIM) mode under positive ionization. For the specific set of amino acids analyzed, the M + 1 *m*/*z* values were monitored and quantified. The separation column was a Hypercarb column with dimensions of 50 mm × 2.1 mm i.d. and a particle size of 3 µm (Thermo Scientific). Column temperature during sample run was 55 °C. Solvents for gradient elution were water with 0.1% formic acid (solvent A) and methanol with 0.1% formic acid (solvent B). The elution program included an isocratic phase from 0 to 2 min with 100% solvent A, followed by a gradient elution from 2 to 5 min decreasing from 100% to 10% solvent A. This was followed by a second isocratic elution from 5 to 9.4 min with 10% solvent A. Column conditioning consisted of an isocratic elution from 9.4 to 14 min with 100% solvent A.

The flow rate was constant at 0.25 mL/min. The analysis time was 14 min. Sample vials were thermostatted at 8 °C. The rinse solution was water with 10% methanol (*v*/*v*). Injection volumes were 5 µL for both the standards and the samples.

### 4.6. Analysis of Selected Phenolic Compounds

Standards for phenolic analysis (chlorogenic acid, rutin, quercetin, quercitrin, isoquercitrin, and hyperoside; Extrasynthese, Genay, France) were prepared by dissolving 5 mg of each standard in 10 mL of methanol (Merck, Darmstadt, Germany). The solutions were then diluted with 50% (*v*/*v*) methanol. With this, we prepared a working standard solution with a concentration of 20 µg/mL for each analyte. The diluted nectar samples were analyzed using a UHPLC Vanquish system (Thermo Scientific, San Jose, CA, USA), equipped with a UV detector (detection wavelengths set at 330 nm and 360 nm) and Chromeleon 7.2 SR4 data acquisition software (Thermo Scientific). The separation column was a Hypersil Gold C18 column with dimensions of 100 mm × 2.1 mm i.d. and particle size of 3 µm (Thermo Scientific) at 40 °C. The solvents for the gradient elution were water with 0.1% formic acid (solvent A) and acetonitrile with 0.1% formic acid (solvent B). The elution program included an isocratic elution from 0 to 7 min with 10% solvent B, followed by a gradient elution from 7 to 14 min increasing from 10% to 60% solvent B. Column conditioning consisted of an isocratic elution from 14.1 to 19 min with 10% solvent B. The flow rate was constant at 0.25 mL/min. The analysis time was 19 min. The sample vials were thermostatically set at 10 °C. The cleaning solution was water with 10% methanol (*v*/*v*). The injection volumes were 5 µL for standard solutions and 10 µL for samples.

### 4.7. Statistical Analysis

The recorded chromatograms from the HPLC analysis were first evaluated, and based on the standards, the concentrations of the analyzed compounds (sugars, phenolic compounds, and amino acids) were calculated. These concentrations had to be converted to pre-dilution concentrations. The recalculated concentrations were then divided by the number of flowers sampled on each individual plant. This yielded the concentration of the analyzed compounds per individual flower. Basic data processing was performed in Excel (to obtain the mean values of individual compounds). To examine the effect of time of day on the concentration of selected compounds, we first applied the Kolmogorov–Smirnov test and determined that the data were not normally distributed. Statistically significant differences between the different times of day and the concentrations of the analyzed compounds were first examined using the Kruskal–Wallis test. If the test indicated the presence of statistically significant differences, we further used the Mann–Whitney U test to determine exactly between which times of day these differences occurred and for which compound concentrations. Graphs showing the effect of time of day on compound concentrations were created in SPSS 23.0.0.0, while the others were generated in Excel.

## 5. Conclusions

In *Fragaria vesca*, the highest amounts of nectar secreted were observed at 9:00, and the lowest were observed at 15:00, when frequently nectar sampling was not possible due to low secretion. On average, *F. vesca* secreted 0.02 μL of nectar per flower throughout the day. The sugar composition of the nectar is hexose-dominant, with a very low proportion of sucrose. The dominant phenolic compound was rutin, while chlorogenic acid was the least abundant. Among amino acids, proline was the most abundant, and methionine was present in the lowest concentrations. We found that the time of sampling significantly influenced all measured nectar parameters: the secreted volume and the concentrations of individual compounds (sugars, amino acids, and phenolic compounds). The maximum values of these parameters were found at different times of day. While the nectar volume as well as the concentration of amino acids and phenolic compounds reached their maximum in the morning, sugar concentrations peaked at noon. Given that *Fragaria vesca* is a common species in nature in Slovenia—known to many for its tasty fruit—our research demonstrates that it is also an excellent forage plant for bees and other pollinators, providing not only pollen but also nectar. Even if *F.vesca* secreted the most nectar in the morning, the concentration of its compounds was higher at noon, which is especially useful for bees in the springtime, as they are not as active in the mornings as at noon. In terms of nutritional value, the quality is more important than the amount of nectar. The greater amount of secreted nectar in the morning could also be due to the higher humidity in the morning, as the plants were wet. A study in a controlled environment would probably yield even more accurate results regarding nectar characteristics. Our study was conducted in nature because we wanted to find out what the characteristics of nectar are in a natural environment, where we did not have to control for the abiotic factors that also affect nectar.

## Figures and Tables

**Table 1:** Average mass of the analyzed compounds per flower of *F. vesca* in sampled nectar.

Analyzed Compound	Mass (ng)
Sucrose	100
Glucose	500
Fructose	500
Chlorogenic acid	0.3
Rutin	0.9
(Iso)quercitrin	0.6
Hyperoside	0.5
Quercetin	0.5
Proline	1.4
(Iso)Leucine	0.13
Methionine	0.02
Alanine	0.56
Tyrosine	0.14

**Table 2:** Average concentrations of analyzed sugars in the nectar of a single flower of *F. vesca* throughout the day.

Time	Sucrose (mg/mL)	Glucose (mg/mL)	Fructose (mg/mL)
09:00	0.50	2.86	2.75
12:00	0.21	4.44	4.05
15:00	0.16	1.34	1.71

**Table 3:** Average concentrations of analyzed aminoacids in the nectar of a single flower of *F. vesca* throughout the day.

Time	Proline (mg/mL)	(Iso)leucine (mg/mL)	Methionine (mg/mL)	Alanine (mg/mL)	Tyrosine (mg/mL)
09:00	0.0491	0.0028	0.0003	0.0035	0.0015
12:00	0.0121	0.0006	0.00010	0.0011	0.0012
15:00	0.0061	0.0013	0.00012	0.0010	0.00107

**Table 4:** Average concentrations of analyzed phenolic compounds in the nectar of a single flower of *F. vesca* throughout the day.

Hour	Chlorogenic Acid (mg/mL)	Rutin (mg/mL)	(Iso)quercitrin (mg/mL)	Hyperoside (mg/mL)	Quercetin (mg/mL)
09:00	0.001	0.017	0.013	0.010	0.002
12:00	0.010	0.020	0.007	0.003	0.014
15:00	0.0001	0.0006	0.001	0.002	0.003

## Data Availability

The original data presented in the study are openly available in the repository of University Botanic Gardens Ljubljana, Biotechnical faculty.
